# Clinical and Demographic Characteristics Including Comorbidities and Their Outcomes Among Patients Hospitalized With COVID-19 in Four Tertiary Care Hospitals Across Lahore

**DOI:** 10.7759/cureus.12663

**Published:** 2021-01-12

**Authors:** Aijaz Zeeshan Khan Chachar, Khurshid Khan, Asma A Khan, Khan Muhammad Imran Hasan, Muhammad Ashraf Zia, Nasir Siddique, Bilal Bin Younis, Zohaib Ahmad Khan

**Affiliations:** 1 Medicine, Fatima Memorial Hospital College of Medicine and Dentistry, Lahore, PAK; 2 Medicine and Endocrinology, Fatima Memorial Hospital College of Medicine and Dentistry, Lahore, PAK; 3 Internal Medicine, Ameer-ud-Din Medical College, Lahore, PAK; 4 Anaesthesia and Critical Care, Jinnah Hospital, Lahore, PAK; 5 Medicine, Ganga Ram Hospital, Fatima Jinnah Medical College, Lahore, PAK; 6 Internal Medicine, Shaikh Zayed Hospital, Lahore, PAK

**Keywords:** covid-19, sars-cov-2, comorbidities, outcomes, demographics, coronavirus

## Abstract

Background

The first case of infection with severe acute respiratory syndrome coronavirus 2 (SARS-CoV-2) was diagnosed in Wuhan, China, in 2019. By the first half of 2020, coronavirus disease 2019 (COVID-19) turned into a global pandemic.

Objectives

The aim of this study is to describe the clinical and demographic characteristics including comorbidities and their outcomes among patients hospitalized with COVID-19 in four tertiary care hospitals across Lahore*. *This retrospective study was conducted at Fatima Memorial Hospital, Sir Ganga Ram Hospital, Lahore General Hospital, and Jinnah Hospital, all in Lahore, Pakistan, from May 1, 2020, to June 30, 2020. The sample size was 445, which was derived using the convenient sampling method. Clinical outcomes during hospitalization included the requirement of invasive positive pressure ventilation, need for renal replacement therapy (RRT), and death. Data regarding demographics, baseline comorbidities, important vital signs on reporting, and initial workup with results were also collected.

Results

A total of 445 patients’ data were studied, of whom 291 (65.4%) were male patients and 154 (34.6%) female patients. The median age was 54 years (interquartile range [IQR]: 24). The most common comorbidities were hypertension (HTN) (195; 43.8%) followed by diabetes mellitus (DM) (168; 37.8%) and cardiovascular disease (CVD) (61; 13.7%). The median length of hospital stay was eight days (IQR: 3). Of the total patients, 137 (30.7%) were treated in intensive care unit settings, 40 (9%) received invasive mechanical ventilation, 40 (9%) patients had acute kidney injury, 38 (8.5%) received RRT, and 37 (8.3%) died. It was seen that more patients who were either diabetic or hypertensive received invasive mechanical ventilation as compared to those who did not have these comorbidities. The most common radiological finding on chest X-ray was the classical ground-glass appearance of COVID-19, which was found in 318 (71.4%) patients.

Conclusions

Patients with one or more underlying comorbidities had poor clinical outcomes compared to those with no comorbidities, with the most vulnerable group being patients with chronic kidney disease, DM, HTN, and CVD in descending order.

## Introduction

The first case of infection with severe acute respiratory syndrome coronavirus 2 (SARS-CoV-2) was diagnosed in Wuhan, China, in 2019. By the first half of 2020, coronavirus disease 2019 (COVID-19) turned into a global pandemic, having different forms of presentation in different patients. The exponential rise in its cases along with associated mortality has shaken the world [1].

The SARS-CoV-2 is a single-stranded RNA virus that results in severe acute respiratory syndrome. The World Health Organization (WHO) declared SARS-CoV-2 as COVID-19 and also a global health emergency. The first diagnosed case of infection was reported at the start of December 2019 and consequently invaded different countries including Europe and the United States [2,3].

Generally, 75% of patients recover without any notable complication; however, 25% can experience associated complications leading to intensive care unit (ICU) transfer and even mortality [4].

More than 210 countries have been affected, with more than 1.8 million confirmed cases of COVID-19 and more than 110,000 deaths. In regard to recovery of the patients, over 412,000 people have come out of this deadly disease [5]. The procurement rate has significantly risen because of the panic created in the public, and educational, religious, and political gatherings have been postponed so as to observe and comply with directions to the countries to implement the preventive policies issued by the WHO.

Pakistan is already going through a tough phase of economic rejuvenation and having limited resources to deal with COVID-19. Pakistan officially declared the first case of this devastating disease in the country in February 2020 [5].

Comorbidities are frequently seen in COVID-19 patients. On presentation, around half of patients had one comorbidity, with diabetes mellitus (DM) and hypertension (HTN) in 20% with the most common being other cardiovascular and cerebrovascular diseases (7-40%) [6-8]. These patients are more vulnerable to develop respiratory failure, which can also result in higher mortality in these cases [7,9].

HTN and ischemic heart diseases came out to be the most common group of comorbidity [6-9]. Among endocrine diseases, DM tops the list in patients infected with COVID-19.

A study conducted by Kulcsar et al. [10] found that the Middle East respiratory syndrome (MERS-CoV) infections ended up in prolonged inflammation in the airways along with carrying dysfunctional immune cells and a modified expression profile of inflammatory mediators in diabetic mice models. Going through a literature search, it was found that SARS-CoV infections led to immune system dysfunction, ultimately causing cardiac diseases, bone diseases, and malignancy [11]. Therefore, a disturbance in the immune system and chronic inflammation may have dominant roles in predicting the unfavorable clinical outcomes in patients with COVID-19, but future research and results of clinical trials will be a source of a great learning curve for all of us.

No specific effective antiviral treatment has been approved worldwide yet in the current armamentarium for COVID-19. Optimized supportive care remains the mainstay of therapy. As of now, more than 300 clinical trials are ongoing, various antiviral and immunomodulating agents are in different stages of evaluation for COVID-19 in those trials, and results/primary endpoints of few trials will be published in the next couple of months.

However, previous studies conducted had few limitations in their study designs, including the relatively small sample sizes and single-center observations.

Our study is the largest retrospective multicenter study in Lahore to find out the impact of comorbidities on the clinical characteristics and prognosis in patients with COVID-19 in four major tertiary care hospitals in Lahore.

## Materials and methods

Objectives

The aim of this study was to assess the clinical and demographic characteristics including comorbidities and their outcome among patients hospitalized with COVID-19 in four tertiary care hospitals across Lahore.

Operational definition

COVID-19 Infection

Patients were labeled to have COVID-19 who had SARS-CoV-2 (previously named as 2019-nCoV) proven via typical clinical features of COVID-19 and typical radiological findings along with positive COVID-19 polymerase chain reaction (PCR) test or negative PCR test.

Acute Kidney Injury

Patients were labeled to have acute kidney injury (AKI) if their serum creatinine increased from the baseline by 0.3 mg/dL or more within 48 hours or if their serum creatinine increased 1.5 times of the baseline.

Obesity

Patients were labeled to have obesity if their body mass index (BMI) was ≥ 27.5 kg/m^2^.

Methodology

This is a retrospective study. COVID-19 patients’ data were collected from four different tertiary care hospitals across Lahore, Pakistan: Fatima Memorial Hospital, Sir Ganga Ram Hospital, Lahore General Hospital, and Jinnah Hospital. Our sample size was 445 patients. The duration of the study was from May 1, 2020, to June 30, 2020.

Records of the patients admitted to the four hospitals were studied, and data related to epidemiological, demographic, clinical, comorbidities, treatment, and outcomes were collected and analyzed. All the data were entered on a structured proforma.

Data were entered and analyzed using SPSS 25.0 (IBM Corp., Armonk, NY, USA). Frequency and percentages were calculated for the qualitative variables such as gender, comorbidity, symptoms, response to treatment, and outcomes. Quantitative variables of the study, such as age, were expressed as median (interquartile range [IQR]). Chi-square test was applied, as appropriate.

Patients were included in the study after obtaining Institutional Review Board approval from the Ethical Committee.

## Results

A total of 445 patients’ data were studied from four major tertiary care hospitals in Lahore city, the capital of the biggest province in Pakistan. Of the patients, 291 (65.4%) patients were male and 154 (34.6%) were female. The median age was 54 years (IQR: 24). The most common comorbidities were HTN 195 (43.8%) followed by DM 168 (37.8%) and cardiovascular disease (CVD; 61 [13.7%]). The PCR for COVID-19 was positive in 428 (96.2%) and negative in 17 (3.8%). The median oxygen saturation at presentation was 90% (IQR: 5), and the median oxygen requirement was 10 liters (IQR: 6) (Table 1).

**Table 1:** Baseline characteristics, presentation vitals, and laboratory results of patients hospitalized with COVID-19 IQR, interquartile range; PCR, polymerase chain reaction; CRP, C-reactive protein

Demographics and comorbidities	
Age, median (IQR)	54 (24)
Gender, no. (%)	
Male	291 (65.4)
Female	154 (34.6)
Comorbidities, no. (%)	
Diabetes mellitus	168 (37.8)
Hypertension	195 (43.8)
Cardiovascular disease	61 (13.7)
Chronic kidney disease	29 (6.5)
Obesity	51 (11.5)
Lung disease	54 (12.1)
Smoking	80 (18.0)
Malignancy	9 (2.0)
Oxygen saturation, median (IQR)	90 (5)
Oxygen requirement, median (IQR)	10 (6)
PCR-positive COVID-19 patients, no. (%)	428 (96.2)
Total patients, no. (%)	445 (100)
Triage vitals, no. (%)	
Febrile	173 (39)
Respiratory rate > 24 breaths/minute	89 (20)
Supplemental oxygen	291 (65.4)
Oxygen saturations, no. (%)	
<90%	291 (65.4)
>90%	154 (34.6)
Initial laboratory measures, median (IQR)	
Total leucocytes count	8 (5.80)
Neutrophils	9.6 (65.9)
Lymphocytes	2.8 (12.6)
CRP	72 (71)
Ferritin	653 (471)
D-dimers	678 (781)
Trop-I (troponin above test-specific upper limit of normal)	5.9 (43.7)
Serum creatinine	0.9 (0.6)
Length of stay for patients in hospital at the study end point, days, median (IQR)	8 (3)

At triage, 173 (39%) patients were febrile, 89 (20%) patients had a respiratory rate of >24 breaths per minute, and 291 (65.4%) patients received supplemental oxygen. The median duration of stay in the hospital for patients was eight days (IQR: 3) (Table 1).

Table 2 shows the distribution of discharged patients admitted with COVID-19 by 15-year age intervals, as well as the duration of stay for those patients who died and those who recovered.

**Table 2:** Discharged patients’ distribution hospitalized with COVID-19 by 15-year age intervals

Patients discharged alive or dead at the study endpoint								
	Died, no. (%)			Length of stay among those who died, days, median (IQR)	Discharged alive, no. (%)			Length of stay among those discharged alive, days, median (IQR)
Age	Male	Female	Total		Male	Female	Total	
Up to 30 years	0 (0)	1 (100)	1 (100)	7	29 (72.5)	11 (27.5)	40 (100)	7 (3)
31-45 years	5 (100)	0 (0)	5 (100)	5 (4.5)	73 (67)	36 (33)	109 (100)	7 (3)
46-60 years	4 (44.4)	5 (55.6)	9 (100)	8 (4)	86 (66.2)	44 (33.8)	130 (100)	8 (5)
61-75 years	8 (88.9)	1 (11.1)	9 (100)	8 (10)	62 (60.8)	40 (39.2)	102 (100)	8 (4)
Above 75 years	6 (46.2)	7 (53.8)	13 (100)	8 (4.5)	18 (66.7)	9 (33.3)	27 (100)	9 (4)
Total	23 (62.2)	14 (37.8)	37 (100)	8 (4.5)	268 (65.7)	140 (34.3)	408 (100)	8 (3)

Patients’ outcomes: discharged or died

Among 445 patients studied, 137 (30.7%) were treated in ICU settings, 40 (8.98%) received invasive mechanical ventilation, 40 (9%) patients had AKI, 38 (8.5%) received renal replacement therapy (RRT), and 37 (8.3%) died (Table 3).

**Table 3:** Clinical variants and outcomes for patients discharged alive and dead at the study end point by age ICU, intensive care unit

Clinical measures and outcomes	Discharge			Died		
	Less than 40 years of age	41-65 years of age	Above 65 years of age	Less than 40 years of age	41-65 years of age	Above 65 years of age
Invasive mechanical ventilation	0 (0)	1 (12.5)	2 (9.5)	0 (0)	4 (18.9)	30 (81.1)
Acute kidney injury	6 (6.2)	15 (7)	11 (11.5)	2 (33.3)	4 (18.2)	2 (22.2)
Renal replacement therapy	1 (1.0)	0 (0)	0 (0)	6 (100)	22 (100)	9 (100)
Length of stay, days, median (IQR)	7 (3)	8 (4)	8 (4)	4.5 (7.5)	8 (4)	9 (9)

Of the total 445 patients, 40 (8.98%) required mechanical ventilation, and of those 40 patients, only 3 (7.5%) patients recovered while 37 (92.5%) died. The mortality rate in those patients who did not receive invasive mechanical ventilation and were in the age group of 41-65 years was 7.1%, whereas the mortality rate in those older than 65 years was 7.4%. The mortality rate in those patients who received invasive mechanical ventilation and were in the age group 41-65 years was 87.5% and in those older than 65 years it was 90.5% (Table 3).

Patients’ outcomes with regard to age and comorbidities

Of the total patients, the percentage of the patients who received invasive positive pressure ventilation was higher for the age group older than 65 years as compared to the age group of 41-65 years.

Among the patients who died, a higher number of those who received invasive mechanical support had DM and HTN as compared to other patients who did not have these comorbidities.

Mortality in patients with chronic kidney disease was 7 (24.1%) followed by 23 (13.9%), 7 (11.5%), and 20 (10.39%) for those with DM, CVD, and HTN, respectively (Table 4).

**Table 4:** Association of comorbidities with outcome

Comorbidity	Outcome		
	Recovery	Death	Total
Diabetes mellitus	145 (86.1)	23 (13.9)	168 (37.8)
Hypertension	175 (89.7)	20 (10.3)	195 (43.8)
Cardiovascular disease	54 (88.5)	7 (11.5)	61 (13.7)
Chronic kidney disease	22 (75.9)	7 (24.1)	29 (6.5)
Obesity	49 (96.1)	2 (3.9)	51 (11.5)
Lung disease	49 (90.7)	5 (9.3)	54 (12.1)
Smoking	72 (90)	8 (10)	80 (18.0)
Malignancy	0 (0)	9 (100)	9 (2.0)

X-ray findings

The most common radiological finding on X-ray was the classical peripheral ground-glass pattern of COVID-19, which was found in 318 (71.4%) patients, and rest of the 127 (28.5%) patients had bilateral consolidation or others (Table 5).

**Table 5:** Radiological findings

Classical peripheral ground-glass pattern of COVID-19	Bilateral consolidation and others
318 (71.4%)	127 (28.5%)

## Discussion

This is the first largest multicenter retrospective study from Pakistan conducted on COVID-19 patients to summarize the demographic and clinical characteristics, and their outcome based on the aforementioned variables. COVID-19 patients do have other comorbidities as well. In some studies, around 50% of patients had one comorbidity, 20% had both DM and HTN, and 7-40% had cardiovascular and cerebrovascular diseases [6-8].

These patients are more fragile to develop early complications, such as respiratory failure, which can lead to higher mortality [7,9].

Multiple studies have revealed that HTN and ischemic heart diseases were the most commonly observed group of comorbidities [6-9] and that DM ranks the highest in order in patients having COVID-19.

In our study, circulatory diseases, such as HTN and coronary heart diseases, and endocrine diseases, such as DM, came out to be the most common comorbidities. Patients having one or more underlying diseases showed poorer outcomes than patients with no preexisting disease, as the former have a weak response to resist the disease and thus to halt the progression of the disease [6-9].

A study conducted in New York by Richardson et al. [12] described the median age of the patients to be 63 years (IQR: 52-57 years), which is comparable to that in our study (54 years), and the most common comorbidity in COVID-19 patients described by them [12] was HTN, seen in 56.6% patients, followed by DM, seen in (33.8%) patients; in our study, HTN was also the most common comorbidity (43.8%) followed by DM (37.8%), which is again comparable to the study by Richardson et al., and at triage, their study had a respiratory rate of >24 breaths per minute in 27.8% patients and PCR for COVID-19 was positive in 96.8% patients, whereas our study concluded that respiratory rate > 24 breaths/minute was seen in 20% patients and PCR for COVID-19 was positive in 96.2%, Again our results are highly comparable with the study by Richardson et al. [12].

A study conducted by Sanyaolu et al. [13]. found out that HTN (55.4%) was at the top of the list as a comorbidity followed by DM (37.3%) and CVD (12.4%); in our study, the same sequence of comorbidities was found, HTN (43.8%) as a comorbidity followed by DM (37.8%) and CVD (13.7%) patients, and this can be attributed to the higher prevalence of these diseases in our population.

A large retrospective study conducted in Zhejiang, China, by Ye et al. [14] had similar results about the common comorbidities (HTN and DM); our study also showed the same results, and again higher prevalence in both the countries may be the factor for the same findings in both the studies.

In the studies by Almazeedi et al. [15] and Wu et al. [16], 19% and 41.9% female patients with COVID-19, respectively, were admitted, and in our study, we had 34.6% female patients, which is comparable with our regional study too and can be attributed to the fact that in our region, due to social and religious constraints, males mostly likely work outside and thus were more exposed to the environment to catch the virus.

In this study, the median length of the stay in the hospital at the study end point was eight days (IQR: 3), whereas in the study conducted by Richardson et al. it was 4.1 days ( IQR: 2.3-6.8); the study by Almazeedi et al. [15] had a longer median hospital stay of 18 days (IQR: 13-24), and this difference may be attributed to the fact that study conducted by Almazeedi et al. [15] had strict criteria for discharge during the initial days of COVID-19 pandemic, which needed two consecutive negative nasopharyngeal swab for COVID-19 24 hours apart.

Almazeedi et al. [15] concluded that 88.2% patients were discharged home, whereas in our study, 91.7% patients were discharged home, which is highly comparable with our study.

Almazeedi et al. [15] described that ICU care was offered to 3.6% patients and that only 1.7% died, whereas in our study, ICU care was given to 30.7% patients and 8.3% patients died; these differences can be attributed to the fact that the study conducted by Almazeedi et al. [15] had a younger population and also included asymptomatic patients, whereas in our study, we did not admit the asymptomatic cases and there were few younger patients in our study.

A study conducted in Wuhan, China, by Wu et al. [16] concluded that 21.9% patients died and 26.4% received the highest level of ICU care and. Zhou et al. [17] concluded that 28.3% patients were died and 26% received ICU care. In our study, ICU admissions are comparable, but the difference in mortality rate can be attributed to the fact that the studies by Wu et al. [16] and Zhou et al. [17] were conducted in the very early days of COVID-19 pandemic (study conducted on patients admitted or expired till January 31, 2020) when understanding of the disease was not sound and there were no consensus guidelines for its management.

Richardson et al. [12] found that 14.2% patients were given ICU care, 12.2% patients required invasive positive pressure ventilation, 3.2% required RRT, and 21% died. Of the patients requiring mechanical ventilation, 3.3% patients were discharged alive and 24.5% died. Mortality in patients who received invasive mechanical ventilation in the age group 41-65 years and older than 65 years was 76.4% and 97.2%, respectively. In our study, ICU care was given to 30.7%, invasive mechanical ventilation to 7.6%, and RRT to 8.5%, and 8.3% died. The mortality rate in those patients who received invasive mechanical ventilation in the age group 41-65 years was 87.5% and in those older than 65 years was 90.5%.

In another study conducted by Guan et al. [18], 19.3% of those with at least one comorbidity and 28.5% of those with two or more comorbidities died, whereas our study revealed death in 21.62% patients who had one comorbidity, which is very high, and in 62.1% patients with two or more comorbidities, almost three times the percentages than those with one comorbidity. Importantly, we have observed that patients with COVID-19 having two or more comorbidities have poorer outcomes in contrast to patients who had no or single comorbidity. Therefore, both the type of comorbid condition and the number of comorbidities should be taken into consideration when predicting the outcomes in these patients. Patients who died with one comorbidity in the study conducted by Guan et al. [18] and in our study are comparable, but patients who died having two or more comorbidities carry huge difference (28.5% vs. 62.1%), and this huge difference can be attributed to the fact that in our culture, patients have multiple comorbidities that are not under optimal control and patients get to know late about their underlying diseases due to lack of awareness and affordability issues in low socioeconomic class; Multiple comorbidities in a patient multiply the risk of more complications and lead to higher mortality rates.

Limitations

The major limitation to our study was, firstly, its retrospective design, as well as missing of some data due to deletion, and, secondly, as the COVID-19 cases kept on escalating, there was a drastic change in the recommendations regarding treatment and diagnostic criteria about sending the patient home, all of which may have had slight implications on our results. And lastly, the sample size was smaller in regard to the big city of Lahore, but the novelty of the disease outweighed the sample size and it was conducted in one city of the country. Therefore, results cannot be generalized to the whole population of the country unless a few other studies from different parts of the country reveal similar results as is the case in our study.

Recommendations

It is highly recommended that further multicenter larger studies be conducted after gathering data from all over Pakistan for a better understanding of this novel disease locally as regional variations are quite possible and these implications will help the health policymakers to not only devise a plan but also formulate local guidelines and get those implemented so as to prevent the rapid spread of this devastating disease carrying higher mortality in patients with comorbidities than expected due to its novelty.

## Conclusions

Our results show that there is a strong association of clinical outcome in COVID-19 patients with comorbidities, the type of comorbidity, and the number of comorbidities these patients have. The outcome gets worse as the number of comorbidities increase. This study highlights the patient population that is at a higher risk of death from COVID-19 based on their demographics and associated comorbidities in the Pakistani population. The most vulnerable groups for the poor outcome from COVID-19 are patients with CKD, DM, HTN, and CVD, with mortality rates of 24.1%, 13.9%, 11.5%, and 10.39%, respectively. The mortality rate is significantly increased in patients who need invasive mechanical ventilation, which in this study ranged from 87.5% to 90.5% depending on their age group.
